# HasLoss: a novel Hassanat distance-based loss functions for binary classification

**DOI:** 10.3389/frai.2025.1690830

**Published:** 2026-02-10

**Authors:** Ahmad S. Tarawneh

**Affiliations:** Faculty of Information Technology, Department of Data Science, Mutah University, Karak, Jordan

**Keywords:** machine learning, loss functions, neural networks, optimization, distance metrics

## Abstract

**Introduction:**

Loss functions play a critical role in machine learning, particularly in training neural networks for classification tasks. In this work, we establish a theoretical framework for distance-based loss functions by adapting the Hassanat distance for binary classification.

**Methods:**

Through gradient analysis, we prove that Hassanat losses exhibit bounded gradients with finite Lipschitz constants, providing convergence guarantees and robustness to outliers. We formulate six variants with different error sensitivities and validate these theoretical properties empirically. Their effectiveness is evaluated on synthetic datasets and nine real-world datasets, ranging from a few hundred to nearly 48,000 samples, under controlled experimental conditions. A comprehensive comparison is conducted against widely used loss functions, including Binary Cross-Entropy (BCE), Focal Loss, Mean Squared Error (MSE), and L1 Loss.

**Results:**

Results show that the proposed Hassanat-based losses achieve competitive performance across evaluation metrics, with comparable or slightly improved results in calibration, convergence speed (in terms of epochs), precision, recall, F1-score, and AUC on several datasets, while exhibiting notable robustness to outliers and noise. The estimated Floating Point Operations (FLOPs) shows that the wall-clock time difference is due to implementation gap, not algorithmic. Importantly, Cohen's d effect size and confidence interval analyses shows that some of the proposed variants introduce a larger practical effect size than popular loss functions such as BCE.

**Discussion:**

This work establishes both theoretical foundations and empirical validation for distance-based loss functions. The bounded gradient framework with finite Lipschitz constants provides principled optimization guarantees while explaining observed robustness and convergence behavior. This foundation enables systematic development of robust loss functions tailored to specific application requirements.

## 1 Introduction

Neural networks have gained significant prominence in the last decade due to their remarkable ability to solve diverse tasks across multiple domains. These include, but are not limited to, regression modeling, image segmentation, disease classification and detection, as well as fraud and intrusion detection systems. Among these applications, binary classification represents one of the most fundamental and widely studied problems in machine learning.

In binary classification tasks, neural networks learn to distinguish between two distinct class patterns. This capability has enabled breakthroughs in numerous real-world applications such as medical diagnosis (e.g., cancer detection), email filtering (spam detection), and financial security systems (fraud detection). The essence of binary classification lies in differentiating between two classes: typically represented as a positive class (indicating the presence of a target condition) and a negative class (representing its absence).

The successful design of neural networks for such tasks requires careful selection of hyper-parameters. These critical parameters include the network architecture (number of layers, neurons per layer), choice of activation functions, optimization algorithms, learning rates, and—crucially—the loss function. The loss function plays a pivotal role in network training (Abu-Srhan et al., 2022) by quantifying the discrepancy between the model's predictions and the actual targets (Janocha and Czarnecki, 2017). Through iterative optimization, the network aims to minimize this loss, thereby improving its ability to separate the classes effectively.

The selection of an appropriate loss function depends on several key characteristics:

1. Robustness: Sensitivity to noise and outliers in the data
2. Convergence Properties: Speed and stability during optimization
3. Decision Boundary Formation: Efficiency in learning optimal separation between classes
4. Generalization Performance: Ability to maintain high accuracy on unseen data

These factors collectively determine how well a neural network will perform in practical applications, making the choice of loss function a critical consideration in model design.

In this work, we design six new loss functions for binary classification, all derived from the Hassanat distance metric, which exhibits natural robustness to noise and outliers (detailed in the methodology section). We analyze the advantages and disadvantages of each proposed function from both theoretical and practical perspectives, examining their convergence properties compared to commonly used loss functions for the same task. We demonstrate the effectiveness of the Hassanat loss variants through decision boundary visualizations (qualitative analysis) and performance metrics (quantitative analysis) under various scenarios on synthetic datasets. Moreover, the performance of the Hassanat loss variants is tested and compared against other loss functions on nine real-world datasets with varying sizes and characteristics.

## 2 Literature review

Several loss functions have been proposed and used in the practice of binary classification. Binary Cross Entropy (BCE) is one of the most widely adopted loss functions in this field (LeCun et al., 2002; Kohler and Langer, 2025; Rahbar and Taheri, 2025; Yu et al., 2024). The main advantages of this loss emerge when it is combined with the sigmoid activation function, as it robustly measures the divergence between predicted probabilities and binary ground truth labels.

However, some deficiencies of this loss have been discussed in the literature, including its sensitivity to outliers, vulnerability to noisy labels, and its tendency to overestimate the impact of incorrect high- confidence predictions (Elharrouss et al., 2025; Zong et al., 2024; Terven et al., 2025). These limitations make it a less recommended option for imbalanced datasets, prompting the use of a weighted version of BCE, which, however, requires careful weight tuning depending on the task (Cui et al., 2019; Das and Begum, 2025).

A generalization of cross entropy loss and MAE was proposed in Zhang and Sabuncu (2018), demonstrating improved performance on multi-class datasets like CIFAR-10. However, their analysis focuses primarily on multi-class scenarios, leaving a gap in understanding how such generalized loss functions perform specifically in binary classification tasks, particularly regarding convergence speed and probability calibration.

Focal loss was introduced in Lin et al. (2017) to solve the problem of class imbalance, especially in object detection. Mainly, this problem presents a high imbalance due to the portion the detected object represents compared to the background. It works by lowering the weights of easy samples and strengthening the weight for the hard misclassified examples. The problem with this loss is that it requires tuning of the parameters γ and α for each dataset separately. Although it was designed for object detection, it was adopted to be used in other classification tasks (Aljohani et al., 2023; Qiao et al., 2021; Nie et al., 2022).

PolyLoss was introduced as an enhanced alternative to BCE Loss (Leng et al., 2022). It treats the loss function as a linear combination of polynomial terms with learnable or predefined weights. In their paper, the authors show that PolyLoss outperforms both BCE and Focal Loss in tasks such as image segmentation. Although the addressed tasks may appear unrelated to our focus on binary classification of structured data, the findings in Leng et al. (2022) highlight that popular loss functions still have limitations, and new formulations are needed to better handle the complexity of real-world problems.

Mean Squared Error (MSE) and Mean Absolute Error (MAE), sometimes called L1 loss, are generally used for regression tasks. MSE is known for its sensitivity to outliers, in regression and classification tasks, While L1 is less sensitive and was recommended as a replacement of the MSE loss. However, some recent research showed that these, MSE and L1, loss functions can perform well in some scenarios, such as noisy labels (Ghosh et al., 2017), when combined with proper activation function, like sigmoid. Also, other research shows that L1 loss can be generalized for better performance, which makes it outperforming BCE, in certain scenarios (Lee and Whang-Bo, 2023). Others preferred MSE over L1 due to its calibration advantage, in binary classification (Flach, 2019; Canbek, 2023).

The loss functions mentioned in this section and their variants are commonly used for training neural networks despite their defects and limitations, as they demonstrate good performance in practice (Terven et al., 2025). In this paper, we propose new loss functions that show improved empirical results compared to other loss functions discussed in the literature, including efficient convergence (in terms of epochs), competitive accuracy, and better calibration for binary classification tasks, on both synthetic and real-life datasets.

## 3 Methodology

Our methodology centers on adapting the Hassanat distance, originally proposed for similarity measurement, into effective loss functions for binary classification. This includes defining the Hassanat distance and deriving its loss function. We then present an improved formulation designed for more efficient learning and better decision boundary formation. Subsequently, we describe our experimental setup and evaluate the proposed functions on both synthetic and real-world datasets.

### 3.1 Hassanat distance

Hassanat is a robust distance metric proposed in Hassanat (2014) to measure the distance between two feature vectors, *a* and *b*. It is mathematically expressed as in Equation 1:


$$\begin{array}{l} DValue\left(a,b\right)=\overset{n}{\underset{i=1}{\sum }}D\left(a_{i},b_{i}\right) \end{array}$$ (1)

where


$$D(a_{i},b_{i})=\{\begin{array}{ll} 1-\frac{1+\min(a_{i},b_{i})}{1+\max(a_{i},b_{i})} & \text{if }\min(a_{i},b_{i})\geq 0\\ 1-\frac{1+\min(a_{i},b_{i})+|\min(a_{i},b_{i})|}{1+\max(a_{i},b_{i})+|\min(a_{i},b_{i})|} & \text{if }\min(a_{i},b_{i})<0 \end{array}$$ (2)

The expression *D*(*a*_*i*_*, b*_*i*_) was later simplified in Hassanat et al. (2022) to the form shown in Equation 3:


$$D(a_{i},b_{i})=\{\begin{array}{ll} \frac{\left|a_{i}-b_{i}\right|}{1+\max(a_{i},b_{i})} & \text{if }\min(a_{i},b_{i})\geq 0\\ \frac{\left|a_{i}-b_{i}\right|}{1+\max(a_{i},b_{i})+|\min(a_{i},b_{i})|} & \text{if }\min(a_{i},b_{i})<0 \end{array}$$ (3)

In our work, focusing on binary classification, the model outputs ŷ ∈ [0, 1] via a sigmoid activation, ensuring non-negative inputs to the loss. Consequently, we adopt the first case of Equation 3 as our loss function, which we define in Equation 4:


$$\begin{array}{l} L_{\text{Hassanat}}\left(ŷ,y\right)=\frac{\left|ŷ-y\right|}{1+\max\left(ŷ,y\right)} \end{array}$$ (4)

The loss function in Equation 4 exhibits two key characteristics:

1. Asymmetric Treatment of Classes: The loss behaves differently for false positives (*y* = 0, ŷ → 1) and false negatives (*y* = 1, ŷ → 0). Specifically:

For *y* = 1, the loss reduces linearly: $L=\frac{1-ŷ}{2}$ with a constant gradient (∇ = −0.5).

**Figure 1:**
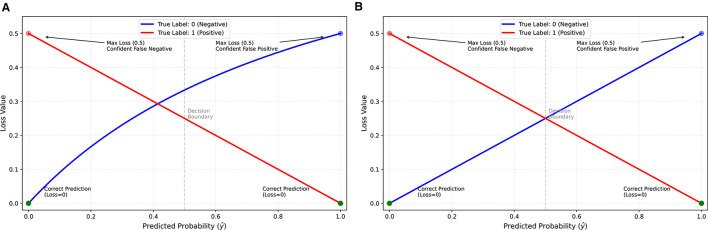
Comparison between the asymmetric Hassanat loss **(a)** and the symmetric variant **(b)**.

For *y* = 0, the loss follows $L=\frac{ŷ}{1+ŷ}$ with a decaying gradient ($\nabla =\frac{1}{{\left(1+ŷ\right)}^{2}}$). This asymmetry can introduce bias during training, particularly in imbalanced datasets, as the model receives stronger correction signals for one class than the other, see Figure 1a.

2. Inconsistent Normalization: The denominator 1+max(ŷ, *y*) scales losses differently for each class. When *y* = 1, the denominator is always 2, whereas for *y* = 0, it varies with ŷ. This inconsistency leads to class-dependent loss scaling, which may bias gradient updates and affect optimization.

These two characteristics can be seen as advantage when the bias toward the negative class is intended, cost-sensitive learning (Fernandez et al., 2018). However, to make formula symmetric we introduce symmetric normalization term as in Equation 5 and Figure 1b.


$$\begin{array}{l} L_{\text{SymHassanat}}\left(ŷ,y\right)=\frac{\left|ŷ-y\right|}{1+\max\left(ŷ,y,1-ŷ,1-y\right)} \end{array}$$ (5)

It is worth noting here that both functions are not differentiable at (ŷ, *y*), because of the absolute value in the numerator. The point of non-differentiability corresponds to an exact match between prediction and target where the loss is zero. This is not a problem in practice for gradient-based optimization methods, since such points are isolated, and subgradient methods or numerical approximations can deal with them. Away from this point, both loss functions are differentiable and thus yield meaningful gradient signals to guide training.

While the symmetric Hassanat loss (Equation 5) achieves balanced gradient magnitudes for both positive and negative classes by normalizing with the term 1+max(ŷ, *y*, 1−ŷ, 1−*y*), this consistency can come at a cost. Specifically, the gradient magnitude under this formulation depends primarily on the normalized absolute difference raised to the first power, which may result in gradients that do not strongly reflect how far the prediction ŷ is from the target *y*. In other words, the symmetric loss yields relatively uniform gradient magnitudes across different error scales, potentially reducing the sensitivity to large deviations. The gradient magnitude with respect to *y* is proportional to


$$\begin{array}{l} \nabla_{ŷ}L\propto \frac{\text{sign}\left(ŷ-y\right)}{1+\max\left(ŷ,y,1-ŷ,1-y\right)} \end{array}$$

which varies slowly with increasing error magnitude |ŷ−*y*|. To address this limitation and amplify the penalization of larger errors, we generalize Equation 5 to the form presented in Equation 6, with *p* represents the power, 1 for linear loss and 2 for quadratic.


$$\begin{array}{l} L_{\text{SymHassanatQuadratic}}\left(ŷ,y\right)=\frac{|ŷ-y{|}^{p}}{1+\max\left(ŷ,y,1-ŷ,1-y\right)} \end{array}$$ (6)

This quadratic form increases the gradient magnitude proportionally to the error size, improving the sensitivity of the loss to predictions further from the target. It preserves the symmetric normalization and boundedness properties while encouraging stronger corrective updates when errors are large.

Another approach to increase the sensitivity of the loss to prediction errors, especially when more aggressive updates are desired, is to apply a logarithmic scaling to the error term 7. This transforms the linear or quadratic penalization into a function that grows rapidly as the prediction approaches the target, effectively emphasizing larger errors.


$${ℒ}_{\text{SymHassanatLog}}(\hat{y},y)=\frac{-\log(1-|\hat{y}-y|+\epsilon )}{1+\max(\hat{y},y,1-\hat{y},1-y)}$$ (7)

where ϵ is a small constant added for numerical stability.

**Figure 2:**
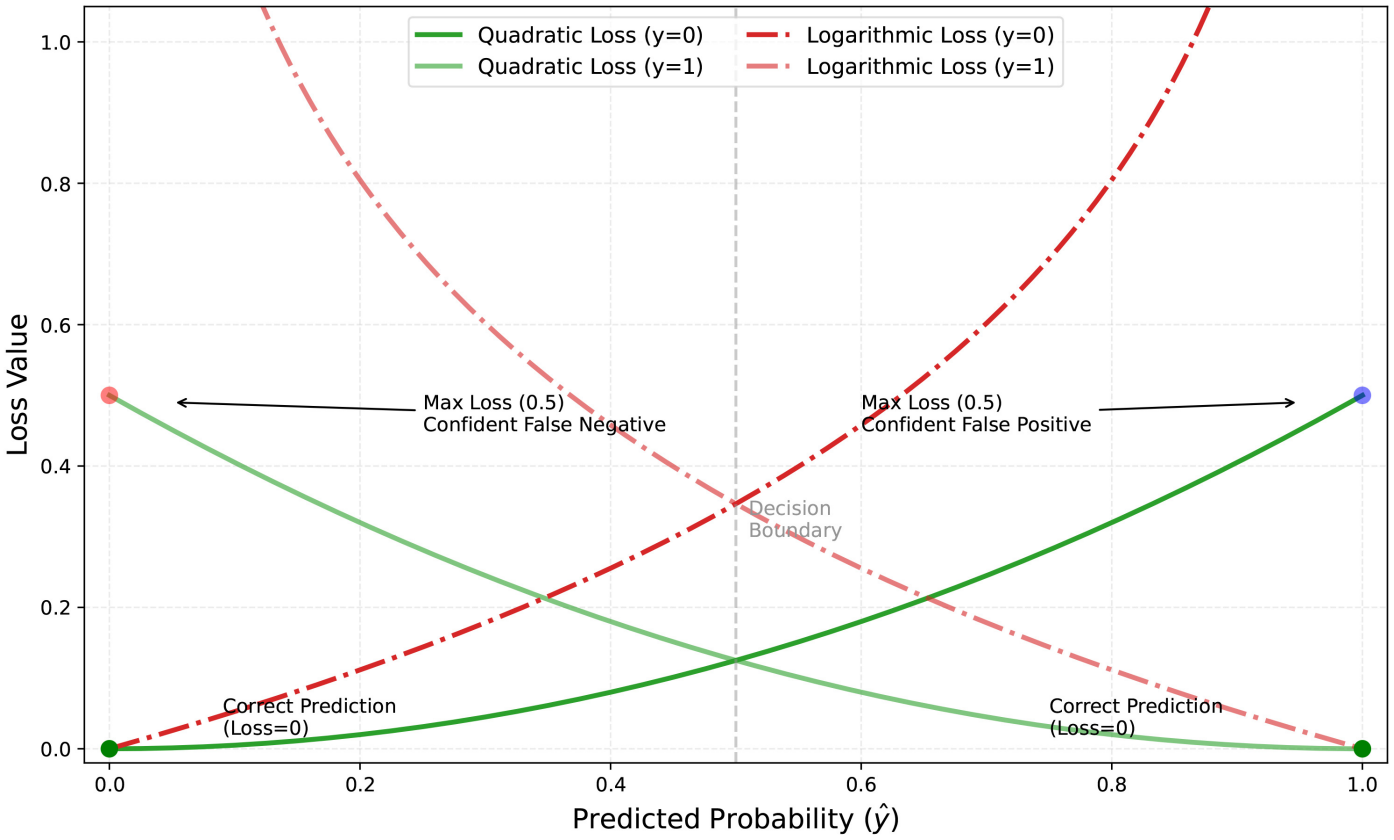
Quadratic and logarithmic variants of the Hassanat loss function.

To prevent extreme values from destabilizing training, the log penalty is clamped at an upper bound. These new variants maintain symmetry and boundedness, while providing stronger gradients near larger errors, encouraging more aggressive corrective updates. Figure 2 visualizes both quadratic and log variants of the proposed loss function.

Scaling the loss based on the normalization expressed in Equations 5–7 provides no explicit awareness of predictions near the decision boundary, the hard cases. These loss variants offer robust and symmetric penalization that effectively bounds error contributions and balances gradient magnitudes across classes, making them well-suited for general classification tasks. However, in scenarios where focusing training effort on ambiguous or borderline predictions is critical, it is advantageous to incorporate a normalization that directly reflects the confidence of predictions relative to the classification threshold. Accordingly, an alternative centrality-based normalization can be used. This normalization term weights the loss inversely proportional to the distance from the midpoint 0.5 for both prediction and target. This refined approach complements the existing formulations by adaptively emphasizing uncertain samples, facilitating sharper decision boundary formation and more efficient convergence. A general formula of the Hassanat loss and its variants using the new normalization term is expressed in Equation 8.


$$\begin{array}{l} L\left(ŷ,y\right)=\frac{\text{Γ}\left(ŷ,y\right)}{\underset{\text{New normalization term}}{\underset{︸}{1+|ŷ-0.5|+|y-0.5|}}} \end{array}$$ (8)

where Γ(ŷ, *y*) denotes the error term, which varies depending on the Hassanat loss variant—for example, the absolute difference |ŷ−*y*|, its square |ŷ−*y*|^2^, or the logarithmic penalty −log(1−|ŷ−*y*|+ϵ).

**Figure 3:**
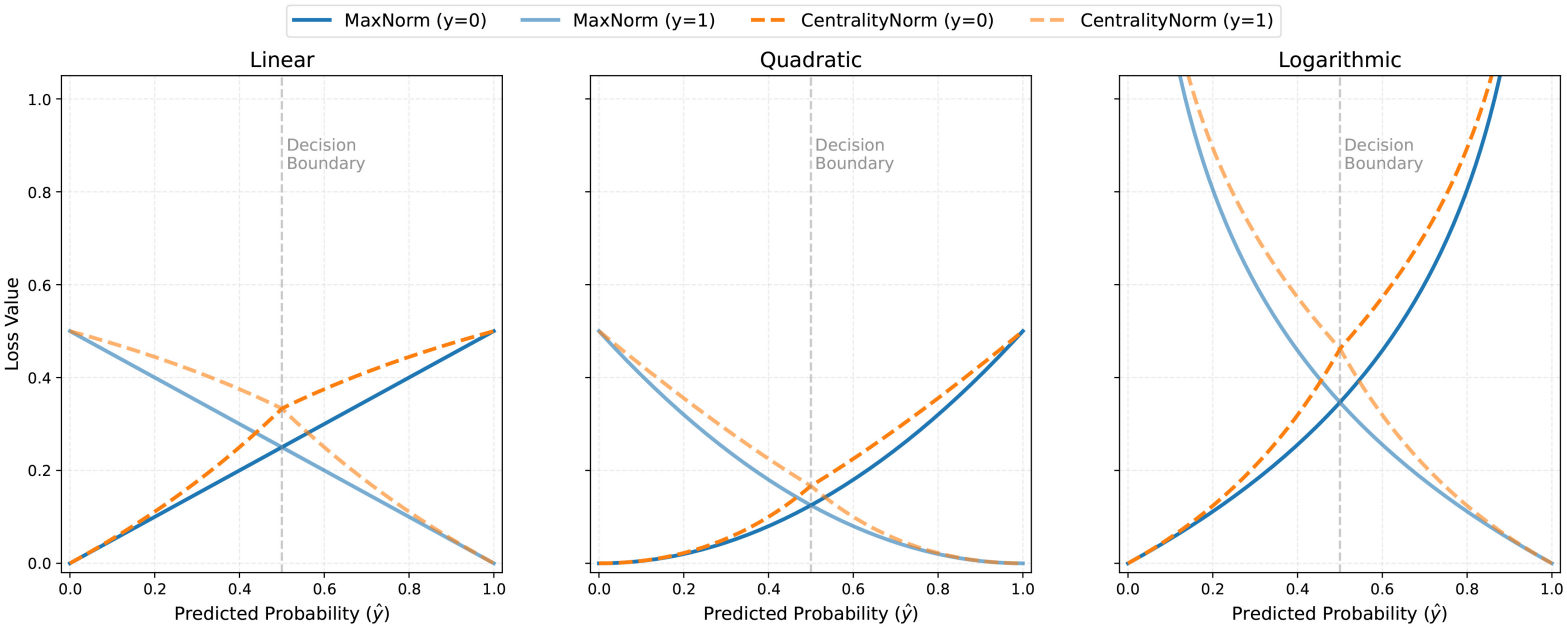
Loss values for the proposed functions using the original and the alternative normalization term mentioned in Equation 8.

This flexible formulation preserves the core error measurement while introducing an adaptive normalization that dynamically scales the loss according to the prediction and target's proximity to the decision boundary. Figure 3 demonstrate the difference between the Hassanat loss values for original and the centrality-based normalization terms.

### 3.2 Theoretical properties and gradient analysis

#### 3.2.1 Motivation for distance-based loss functions

The adaptation of Hassanat distance into loss functions is motivated by three key theoretical properties that address fundamental optimization challenges in binary classification:

Bounded gradient behavior. The Hassanat-based losses exhibit bounded gradients with finite Lipschitz constants. For the symmetric Hassanat loss (Equation 5), let *D* = 1+max(ŷ, *y*, 1−ŷ, 1−*y*)). Since ŷ*, y* ε [0, 1], we have *D* ε [1, 2]. The gradient with respect to ŷ satisfies:


$$\begin{array}{l} |\nabla_{ŷ}L_{\text{SymHassanat}}|\leq \frac{1}{D}\leq 1 \end{array}$$ (9)

This uniform bound holds for all ŷ ε [0, 1] and *y* ε {0, 1}. In contrast, the Binary Cross-Entropy (BCE) loss gradient:


$$\begin{array}{l} \nabla_{ŷ}L_{\text{BCE}}=\frac{ŷ-y}{ŷ\left(1-ŷ\right)} \end{array}$$ (10)

becomes unbounded as ŷ → 0 or ŷ → 1. For instance, with ŷ = 0.99 and *y* = 0, the BCE gradient magnitude is approximately 99, while the Hassanat gradient remains bounded by 0.5. This boundedness prevents gradient explosion and ensures numerical stability during optimization.

**Figure 4:**
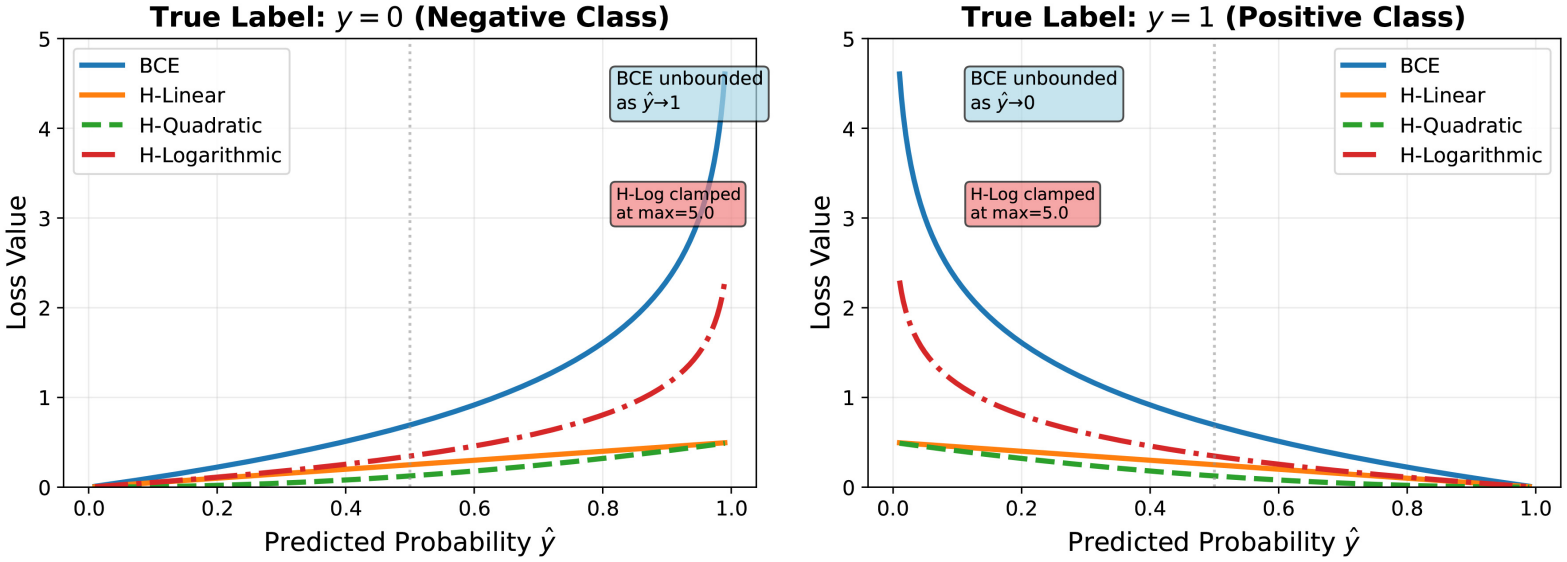
Loss landscape comparison between BCE and Hassanat variants for binary classification. **(Left)** True label *y* = 0 (negative class). **(Right)** True label *y* = 1 (positive class).

Loss landscape geometry. Figure 4 illustrates the geometric differences between BCE and Hassanat loss functions. The key distinction lies in the behavior near confident predictions (ŷ → 0 or ŷ → 1). BCE exhibits unbounded steepness in these regions, creating steep valleys that can destabilize optimization when encountering outliers. In contrast, all Hassanat variants maintain bounded slopes throughout the domain, with H-Linear and H-Quadratic providing the smoothest landscapes (maximum loss = 0.5), while H-Logarithmic offers more aggressive correction while still remaining bounded through clamping (maximum = 5.0). This geometric property ensures that no single sample can produce arbitrarily large gradient updates, providing inherent robustness to label noise and outliers.

Robustness to outliers. The bounded gradient property provides robustness to outliers and label noise. For Hassanat losses, the gradient magnitude is always bounded by |$\nabla_{\hat{\text{y}}}$L(ŷ*, y*)| ≤ 1, which limits the maximum contribution of any single sample to the gradient update. This is similar to M-estimation in robust statistics, where bounded gradients reduce sensitivity to unusual observations. In contrast, unbounded loss functions like BCE can assign very large weights to mislabeled samples, potentially disrupting the optimization process.

Adaptive learning dynamics. The denominator structure in Hassanat losses creates adaptive learning rates that depend on prediction confidence. The effective learning rate scales inversely with *D*, producing larger updates for ambiguous predictions near the decision boundary (ŷ ≈ 0.5) where *D* ≈ 1.5, and smaller updates for confident predictions where *D* ≈ 2. This adaptive behavior focuses optimization effort on challenging samples while maintaining stability for well-classified instances.

#### 3.2.2 Gradient properties of proposed variants

Each variant maintains gradient boundedness while modifying error sensitivity:

Quadratic variant (Equation 6). The quadratic formulation L has error-proportional gradients:


$$\begin{array}{l} \nabla_{ŷ}L_{\text{Quad}}=\frac{2\left(ŷ-y\right)}{D}-\frac{{\left(ŷ-y\right)}^{2}}{D^{2}}\frac{\partial D}{\partial ŷ} \end{array}$$ (11)

with bounded magnitude |$\nabla_{\hat{\text{y}}}$L_Quad_| ≤ 2. This provides stronger correction signals for large errors while maintaining stability.

Logarithmic variant (Equation 7). The logarithmic penalty Γ(ŷ*, y*) = – log(1 – |ŷ – *y*| + ϵ) increases gradient magnitude as errors approach their maximum:


$$\begin{array}{l} \nabla_{ŷ}L_{\text{Log}}=\frac{\text{sign}\left(ŷ-y\right)}{\left(1-|ŷ-y|+\epsilon \right)D} \end{array}$$ (12)

The gradient bound depends on the clamping threshold but remains finite for ϵ > 0.

Centrality-based normalization (Equation 8). The alternative denominator *D*_central_ = 1 + |ŷ – 0.5| + |*y* – 0.5| creates boundary-focused learning. When ŷ≈ 0.5, *D*_central_ ≈ 1.5, maximizing gradient magnitude and emphasizing samples near the decision boundary.

#### 3.2.3 Theoretical comparison with traditional loss functions

Table 1 compares the gradient properties and Lipschitz constants of different loss functions:

**Table 1:** Theoretical properties of loss functions for binary classification.

**Loss function**	**Gradient bound**	**Lipschitz constant**	**Robustness**
BCE	Unbounded	∞	No
MSE	Unbounded	∞	No
Focal Loss	Unbounded	∞	Partial
L1 Loss	1	1	Yes
Huber Loss	Bounded	Finite	Yes
H-Linear	1	1	Yes
H-Quadratic	2	2	Yes
H-Logarithmic	Bounded	Finite	Yes

The finite Lipschitz constants of Hassanat-based losses ensure stable convergence under gradient-based optimization. The loss landscapes do not contain arbitrarily steep regions that could cause training instability or high sensitivity to learning rate selection. This theoretical foundation explains the empirical observations of enhanced robustness and consistent convergence across diverse datasets presented in Section 4.

Connections to optimization theory. The bounded gradient property places Hassanat losses within the framework of Lipschitz continuous optimization, where standard convergence results for gradient descent apply (Nesterov, 2004). For a loss function with Lipschitz constant *L*, gradient descent with step size η ≤ ^1^guarantees convergence. Since Hassanat losses have finite Lipschitz constants (Table 1), these convergence guarantees hold, whereas the unbounded Lipschitz constant of BCE provides no such theoretical assurance. Furthermore, the bounded gradient property aligns with robust optimization principles (Ben-Tal et al., 2009), where the goal is to limit worst-case sensitivity to individual samples. The influence function of Hassanat losses—the gradient with respect to a single sample—is uniformly bounded, placing them in the class of robust M-estimators that have been extensively studied in statistical learning theory (Huber, 2011). This theoretical foundation, combined with empirical validation across diverse datasets, establishes Hassanat losses as principled alternatives to traditional loss functions.

Convexity considerations. Several Hassanat variants maintain convexity: H-Quad is strongly convex (with convexity parameter μ = 1), explaining its superior calibration performance (lowest ECE in Table 4), while H-Linear is piecewise linear and convex. Variants with centrality-based normalization (H-N, H^2^-N) sacrifice global convexity for adaptive boundary focus, achieving superior classification performance through more flexible decision boundaries. The bounded gradient property ensures optimization stability for all variants regardless of convexity structure.

### 3.3 Datasets

Several datasets were used to evaluate the loss functions, comprising both synthetic and real-life data. The characteristics of the real-life datasets are summarized in Table 2. As shown, these datasets vary considerably in the number of observations, number of features, and imbalance ratios. The imbalance ratio is defined as the number of samples in the minority class divided by the number of samples in the majority class; values closer to 1 indicate a balanced class distribution, whereas smaller values reflect a higher degree of imbalance. Across the datasets, the number of observations ranges from approximately 300 to nearly 49,000, while the number of features ranges from 3 to 24.

**Table 2:** Summary of binary classification datasets used in experiments.

**No**.	**Name**	**Features**	**No. observations**	**Imbalance ratio**
1	Adult income prediction	14	48,842	0.315
2	Pima Indians diabetes	8	768	0.536
3	Heart disease	13	270	0.800
4	Credit approval	15	690	0.802
5	Magic gamma telescope	10	19,020	0.542
6	Haberman survival	3	305	0.362
7	Indian liver patient	10	582	0.402
8	Liver disorder	5	345	0.816
9	German credit numeric	24	1,000	0.429

Another set of synthetic datasets with the property described in Table 3. All of the synthetic datasets contains 1000 observations and all are balanced except the first row in Table 3.

**Table 3:** Characteristics of the synthetic data used.

**Dataset name**	**Generation method**	**Noise/corruption**
**Extreme imbalance (5% pos)**	make_classification	None
**Overlapping classes**	make_classification	Class_sep = 0.5
**Non-linear (circles)**	make_circles	Noise = 0.1, factor = 0.3
**Non-linear (Moons)**	make_moons	Noise = 0.15
**With outliers**	make_classification + corruption	30% outliers (σ = 3)
**High feature noise**	make_classification	Gaussian noise (σ = 0.5)

The generation method refers to methods imported from the sklearn module.

### 3.4 Experiments setup

The experiments conducted in this work evaluate the proposed loss functions in comparison to popular alternatives such as Binary Cross-Entropy, Mean Squared Error, and Focal Loss.

All experiments were performed under controlled conditions, where each loss function was tested using the same model architecture initialized with identical weights, ensuring reproducibility and a fair comparison of results. An arbitrary model is chosen, as the primary goal is to compare the relative performance of the loss functions under identical conditions rather than to optimize for the highest accuracy. For the synthetic datasets, we used a simple feed-forward neural network suitable for low-dimensional data. This network consists of two hidden layers with 20 neurons each, activated using ReLU, followed by a single-neuron output layer that produces the predicted logit. For higher-dimensional, real-world datasets, we employed a deep multi-layer perceptron with four hidden layers of sizes 256, 128, and 64 neurons, respectively. Each hidden layer is followed by batch normalization, ReLU activation, and dropout (*p* = 0.2) for regularization, with the final layer producing a single output value.

All experiments were conducted on a Windows Server 2022 (Version 10.0.20348) system equipped with an Intel Xeon processor (32 physical cores, 64 logical threads at 2.5 GHz) and 128 GB DDR4 RAM. The computational workload was accelerated using an NVIDIA RTX A4000 GPU with 16 GB GDDR6 memory (CUDA Driver 555.85) through PyTorch 2.7.0 with CUDA 12.6 backend support. The software environment utilized Python 3.12.4 for all implementations.

Also, the experiments, on synthetic and real-life datasets, are performed using 5-fold cross validation. Exact same random splits are used for all of the tested loss functions to ensure that the difference in performance is due to the selection of the loss function and not any other parameters.

Performance metrics: To comprehensively evaluate the proposed loss functions, we employ multiple performance metrics that assess different aspects of model performance:

Accuracy: Measures the overall correctness of predictions, calculated as:


$$\text{Accuracy}=\frac{\text{TP}+\text{TN}}{\text{TP}+\text{TN}+\text{FP}+\text{FN}}$$

where TP, TN, FP, and FN represent true positives, true negatives, false positives, and false negatives, respectively.

Precision: Evaluates the model's ability to avoid false positives:


$$\text{Precision}=\frac{\text{TP}}{\text{TP}+\text{FP}}$$

Recall (sensitivity): Measures the model's ability to identify all positive instances:


$$\text{Recall}=\frac{\text{TP}}{\text{TP}+\text{FN}}$$

F1-score: The harmonic mean of precision and recall, providing a balanced measure:


$$\text{F1-Score}=2\times \frac{\text{Precision}\times \text{Recall}}{\text{Precision}+\text{Recall}}$$

AUC-ROC: The area under the Receiver Operating Characteristic curve, which evaluates the model's ability to distinguish between classes across all classification thresholds.

Expected Calibration Error (ECE): Quantifies how well the predicted probabilities match the true probabilities, computed by:


$$\text{ECE}=\sum_{i=1}^{M}\frac{\left|B_{i}\right|}{n}\left|\text{acc}(B_{i})-\text{conf}(B_{i})\right|$$

where *M* is the number of bins, *B*_*i*_ contains samples with predicted probabilities in the *i*-th bin interval, acc(*B*_*i*_) is the accuracy of *B*_*i*_, and conf(*B*_*i*_) is the average confidence of *B*_*i*_.

Convergence speed: Measured as the number of epochs required for the training loss to reach and remain within 1% of its asymptotic minimum. Formally, convergence is achieved when:


$$\frac{\left|L_{t}-L_{\text{final}}\right|}{L_{\text{final}}}\leq 0.01$$

for at least *k* = 5 consecutive epochs, where *L*_final_ is the median loss over the final 10% of training. This metric provides a robust measure of optimization efficiency while accounting for training stochasticity.

## 4 Results and discussion

The theoretical analysis in Section 3.2 establishes that Hassanat losses exhibit bounded gradients with finite Lipschitz constants. This leads to three testable predictions: (1) faster convergence on noisy datasets, as bounded gradients prevent extreme updates from outliers; (2) superior decision boundaries on corrupted data, as the bounded landscape prevents overcommitment to mislabeled samples; and (3) best calibration from the strongly convex H-Quad variant, while non-convex variants (H-N) prioritize classification performance. In this section, we validate these predictions on synthetic and real-world datasets under controlled conditions where all loss functions use identical architectures, initialization, and data splits.

### 4.1 Results on synthetic datasets

**Figure 5:**
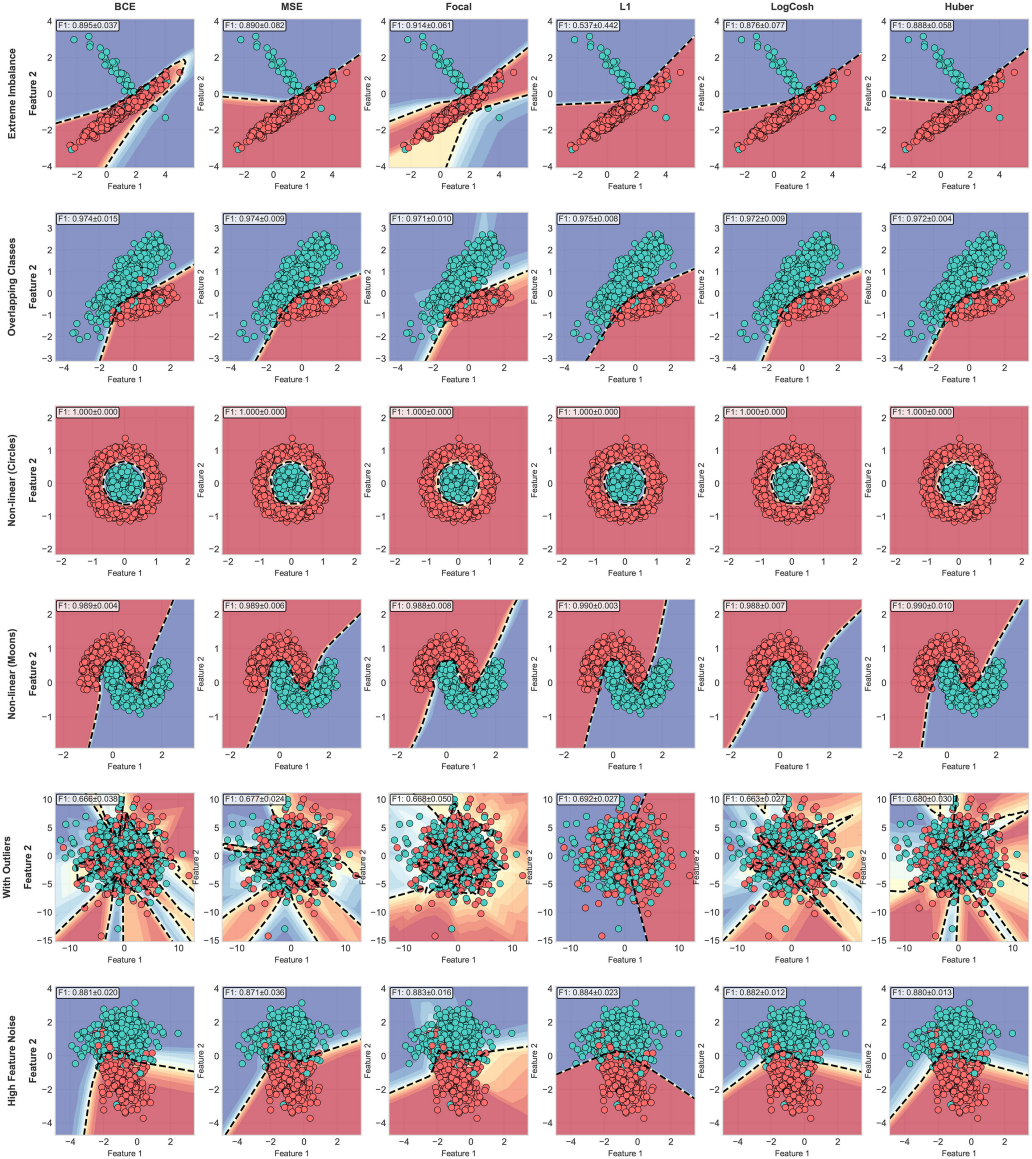
Decision boundaries of Traditional loss functions on all synthetic datasets.

**Figure 6:**
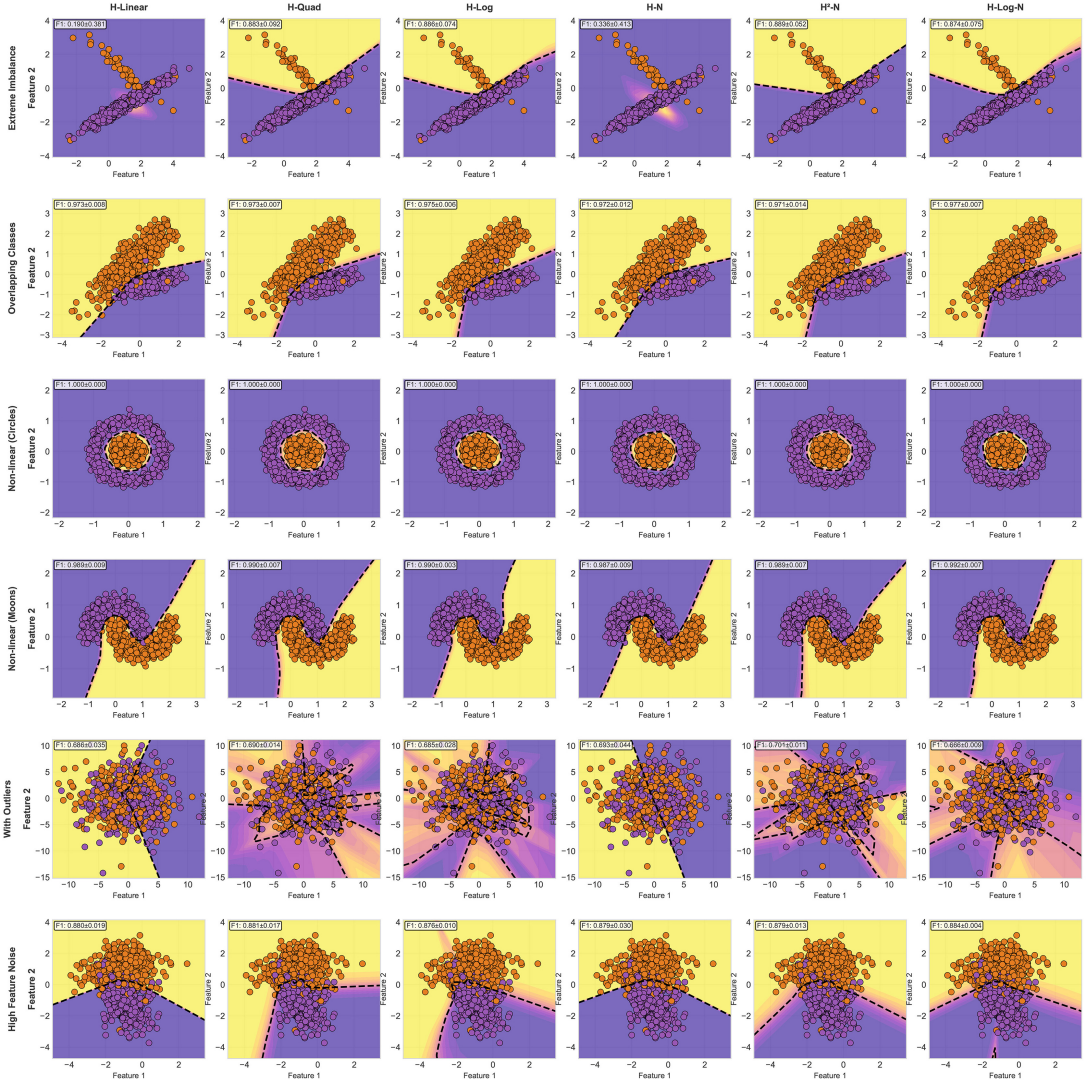
Decision boundaries of Hassanat loss functions on all synthetic datasets.

Figures 5, 6 shows the decision boundaries obtained using each one of the used loss functions on each dataset. As one can see from Figure 5, Focal and BCE loss achieved higher performance than the rest of the functions on the extreme imbalanced dataset. Closer inspection of the data distribution reveals that Focal and BCE achieve this superior performance by being heavily influenced by outlier-like points—samples that appear visually distant from the main cluster of their respective classes. We can see the effect of outliers on their decision boundary on the dataset with outliers, the fifth row from the same figure, while the L1 loss was the least affected by the present outlier despite its strict decision boundaries. On the rest of the datasets the performance was almost similar for all traditional loss functions.

**Figure 7:**
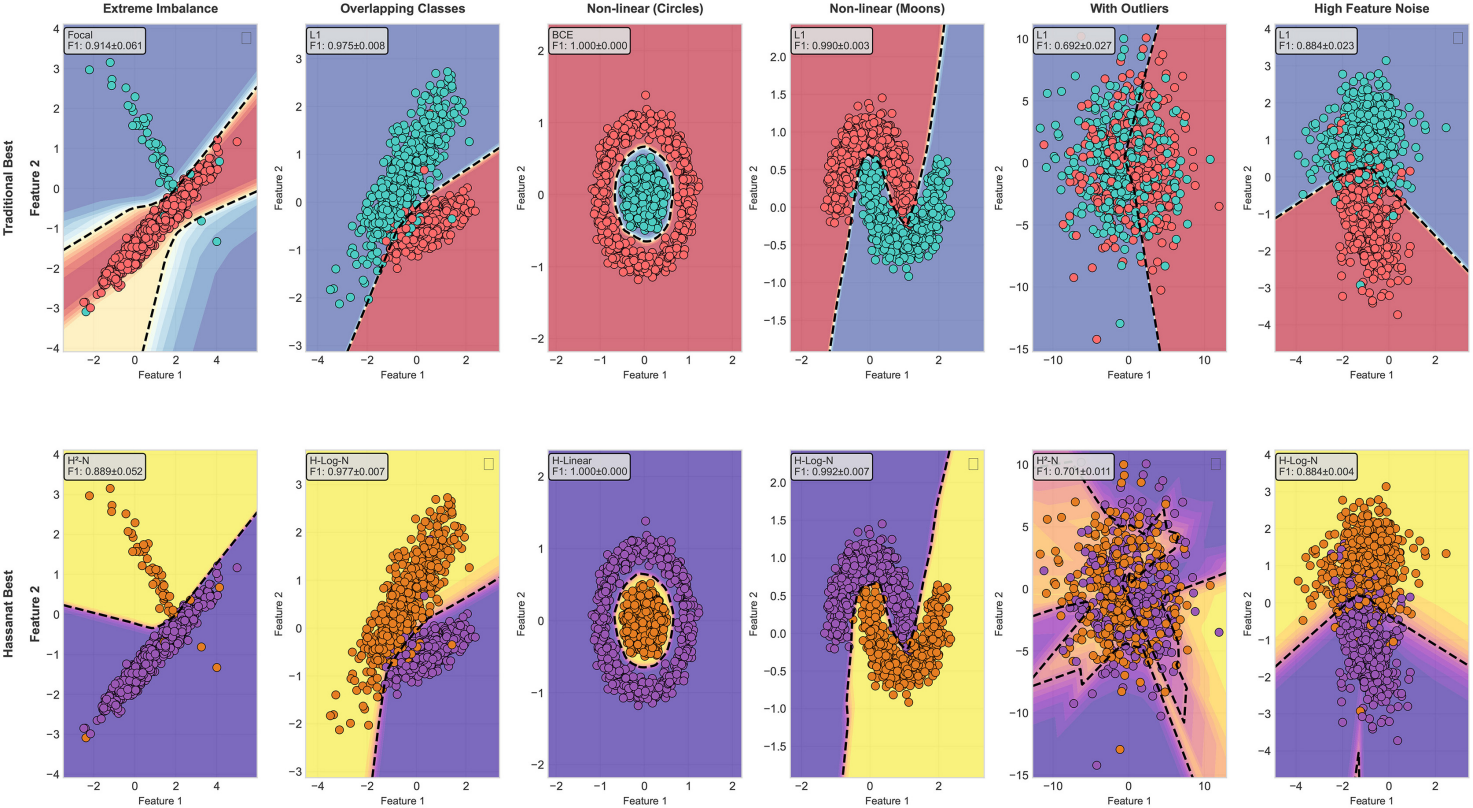
Comparison between the best performers from the traditional and Hassanat loss functions on each synthetic dataset.

To clearly show the difference between the traditional and the Hassanat functions, Figure 7, compares the best from the traditional family to the best from the family families in terms of cross validation F1-score values.

As one can notice, The functions from Hassanat family provides comparable results to the ones in the traditional functions or slightly better, except on the extreme imbalance, the first column in Figure 7, and this due to the fact that Focal loss is influenced by some outlier-like points. On the dataset with outliers, fifth column, we can clearly see that Quadratic Hassanat forms better decision boundaries than the best performer from the traditional functions, L1. The decision boundary gives lower confidence on areas where outliers appear, which led to better F1-score value.

**Figure 8:**
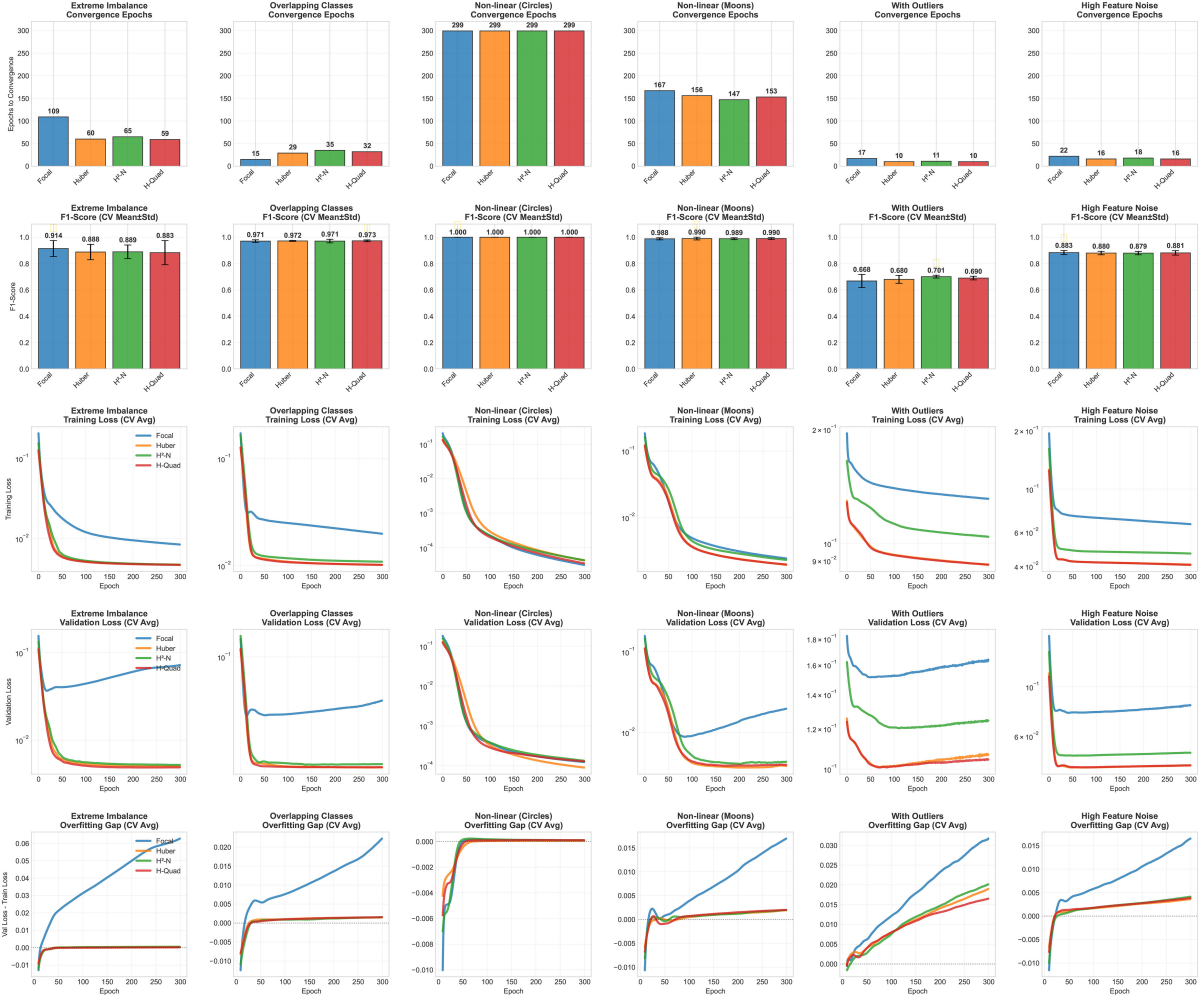
Convergence analysis of the traditional and Hassanat best performing loss functions.

Regarding the convergence, Figure 8 shows the convergence analysis for the best performers from both loss families to converge. Unlike the decision boundaries, where the best performers selected for each dataset, the best performers in Figure 8 is selected based on the mean F1-score across all the datasets. These functions are Focal and Huber functions, from the traditional family, and Quadratic Hassanat (using the maximum and the alternative normalization methods).

The first row in Figure 8, shows the number of epochs needed for convergence. On extreme imbalance dataset, Focal loss takes 109 epochs to converge, while the minimum number of epochs needed for convergence is recorded by Quadratic Hassanat loss. The advantage of Hassanat loss comes into play on both outlier and noisy datasets, where it took only about 10 and 16 epochs to converge, respectively, with a comparative F1-score values. On the last two datasets, we can see from the third and fourth rows of Figure 8 that the training using Huber and Quadratic Hassanat show much better and smoother training convergence. When it comes to Focal loss, although it gives the best F1-score from the traditional family across all datasets, its generalization is poor. This can be seen from the overfitting gap it creates on almost every synthetic dataset used; while Hassanat loss functions with Huber loss, give much better generalization to the validation set.

**Figure 9:**
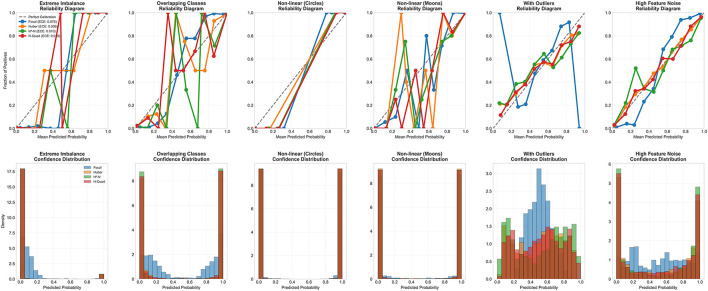
Calibration analysis of best-performing loss functions across six datasets. Top row shows reliability diagrams comparing predicted probabilities with actual outcome frequencies; perfect calibration follows the diagonal line. Bottom row displays confidence distributions of predicted probabilities.

In addition to their robustness on noisy and outlier-prone data, Hassanat quadratic variants exhibit strong calibration and confident predictions. Figure 9 (top row) compares the calibration of the best-performing loss functions across synthetic datasets. The Quadratic Hassanat (H-Quad) loss demonstrates particularly stable calibration under noise and outliers, matching Huber loss in reliability while outperforming Focal loss. Notably, H-Quad achieves superior confidence levels on outlier-contaminated data (fifth column, bottom row of 9), with predictions more tightly concentrated near 0 or 1 compared to both Huber and Focal losses.

Average ECE further support these observations: Huber (0.0182), H-Quad (0.0183), and Focal (0.0640). While Huber and H-Quad are nearly identical in overall calibration, H-Quad excels on outlier-prone datasets (e.g., With Outliers), underscoring its dual advantage of robustness and reliability.

### 4.2 Results on real-life datasets

Table 4 describes the performance for each used loss function across the nine used datasets. As one can see from the results in Table 4, Linear Hassanat with centrality-based normalization (HN) outperforms the rest of the loss functions in terms of Precision, Recall and F1-score values. However, when it comes to calibration, its performance is much lower than the rest. Quadratic Hassanat recorded the best calibration results as it obtained the lowers ECE value across all datasets.

**Table 4:** Comprehensive performance evaluation of Hassanat loss variants and traditional loss functions on nine real-world binary classification datasets.

**Loss function**	**Accuracy**	**Precision**	**Recall**	**F1-score**	**AUC-ROC**	**ECE**
H-N	0.773 ± 0.083	**0.690** **±0.160**	**0.610** **±0.177**	**0.642** **±0.164**	0.781 ± 0.100	0.204 ± 0.072
H-Linear	0.771 ± 0.087	0.681 ± 0.160	0.600 ± 0.187	0.634 ± 0.173	0.792 ± 0.105	0.196 ± 0.071
Huber	**0.774** **±0.086**	0.688 ± 0.161	0.594 ± 0.195	0.633 ± 0.178	0.796 ± 0.116	0.161 ± 0.059
L1	0.769 ± 0.087	0.678 ± 0.165	0.594 ± 0.189	0.629 ± 0.176	0.790 ± 0.105	0.198 ± 0.069
H-Log	0.766 ± 0.087	0.676 ± 0.174	0.590 ± 0.184	0.626 ± 0.176	**0.805** **±0.104**	0.105 ± 0.059
LogCosh	0.770 ± 0.088	0.678 ± 0.180	0.590 ± 0.199	0.626 ± 0.188	0.804 ± 0.108	0.114 ± 0.058
Focal	0.764 ± 0.095	0.674 ± 0.176	0.589 ± 0.193	0.624 ± 0.182	0.796 ± 0.116	0.111 ± 0.025
H-Quad	0.768 ± 0.087	0.677 ± 0.176	0.586 ± 0.199	0.624 ± 0.188	0.804 ± 0.109	**0.102** **±0.054**
H-Log-N	0.768 ± 0.094	0.678 ± 0.171	0.585 ± 0.202	0.622 ± 0.187	0.802 ± 0.111	0.117 ± 0.058
H^2^-N	0.768 ± 0.091	0.674 ± 0.176	0.586 ± 0.204	0.622 ± 0.191	0.801 ± 0.114	0.117 ± 0.056
BCE	0.765 ± 0.092	0.674 ± 0.177	0.585 ± 0.196	0.621 ± 0.185	0.801 ± 0.111	0.103 ± 0.057
MSE	0.763 ± 0.095	0.666 ± 0.183	0.579 ± 0.210	0.615 ± 0.198	0.801 ± 0.111	0.104 ± 0.055

Results show mean ± standard deviation across 5-fold cross-validation. Bold values indicate top-performing methods for each metric.

**Figure 10:**
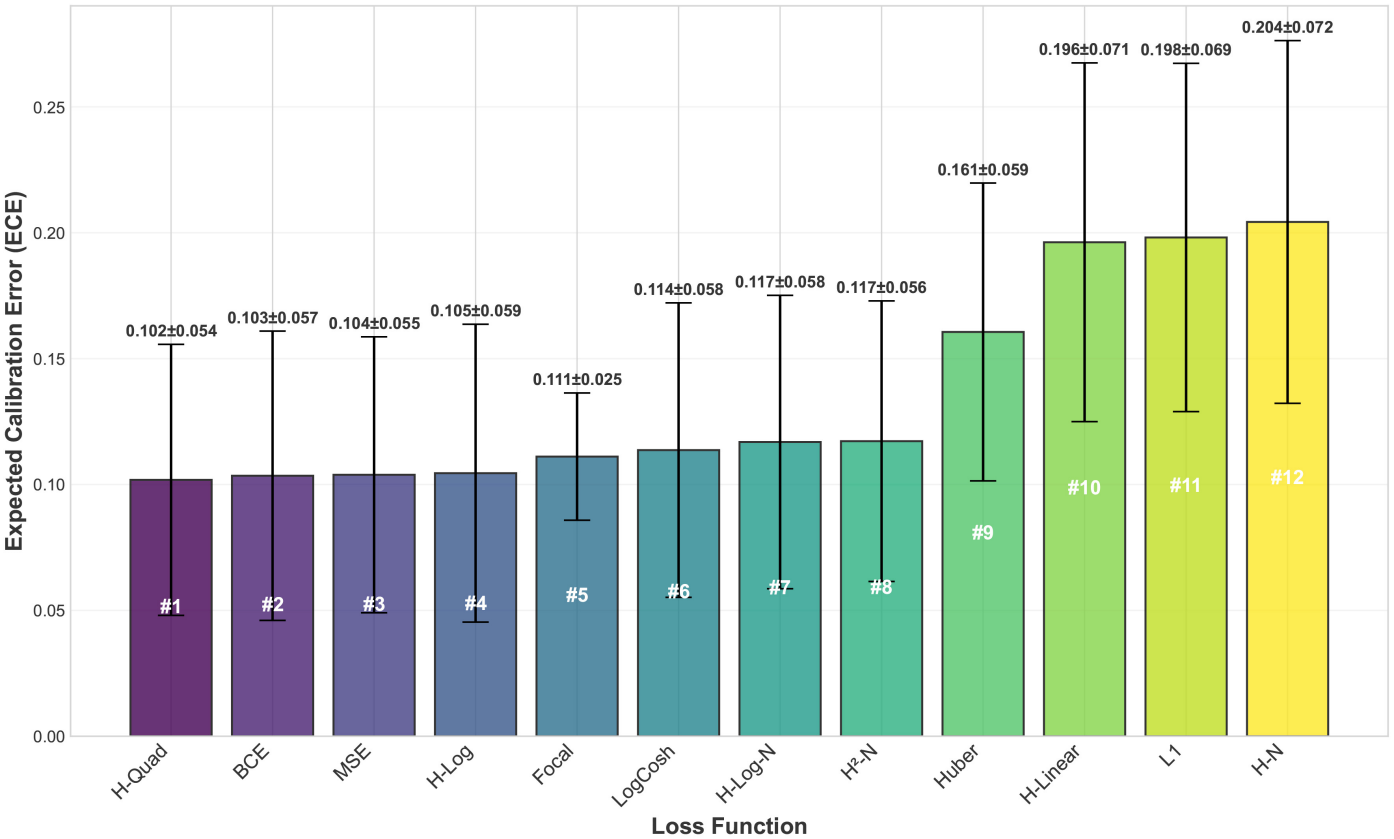
ECE values for the proposed and traditional loss functions averaged across all datasets. The results are sorted from best to worst calibration performance (left to right), where lowest ECE values indicate better calibration.

Figure 10 compares mean ECE values of the tested loss functions across all datasets. As it can be clearly seen, Quadratic Hassanat comes at the first place, with a ECE slightly better than BCE loss function. Although HN recorded best classification performance, it shows poor calibration characteristics compared to other loss functions.

**Figure 11:**
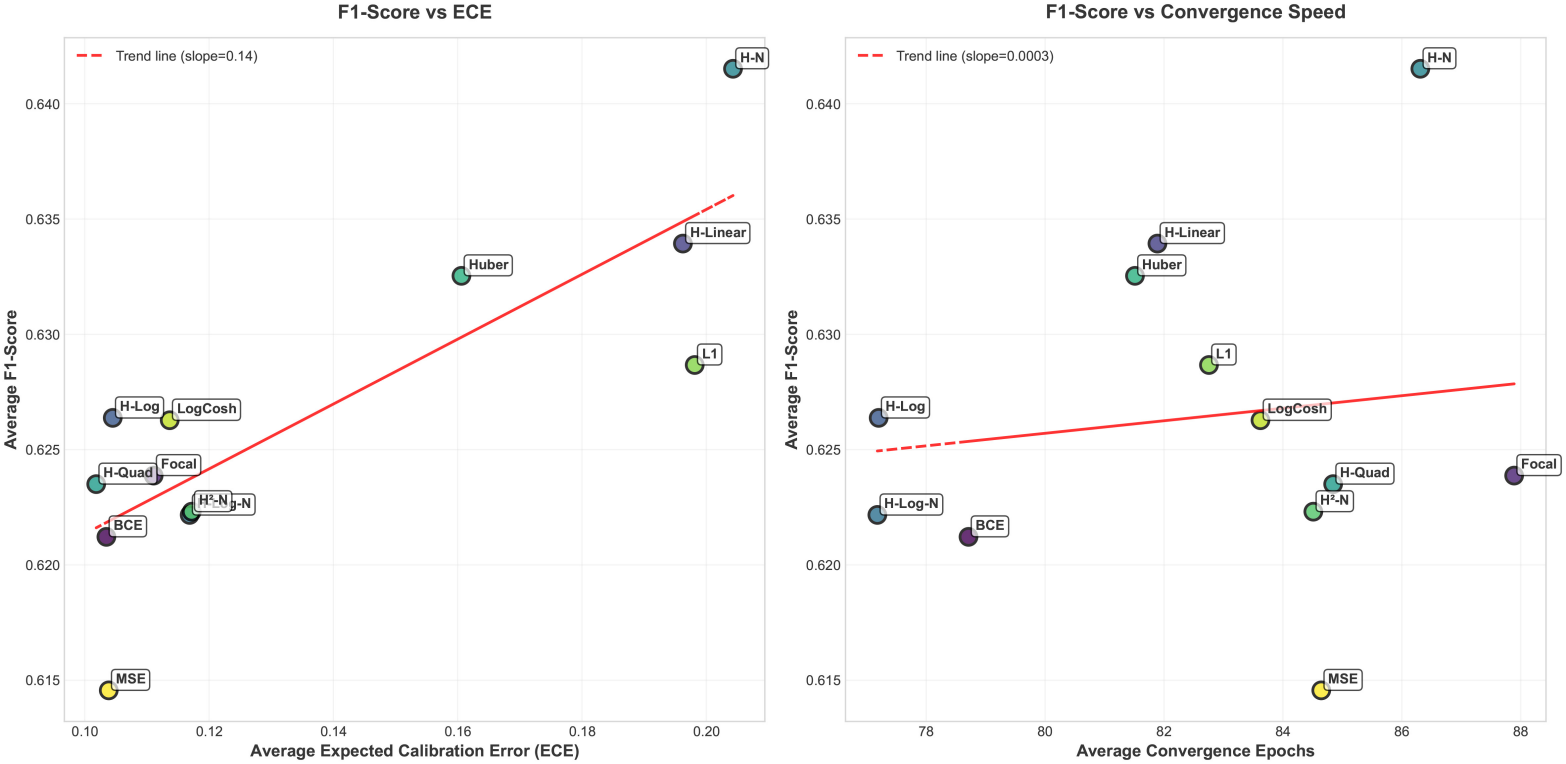
Average calibration and convergence speed, in epochs, of the proposed loss functions across all datasets.

In addition to calibration outcomes, the Hassanat variants exhibit favorable convergence behavior while preserving competitive F1-scores. Figure 11 illustrates the relative ranking of the evaluated loss functions, where the F1-score is analyzed as a function of the ECE and the average number of epochs required for convergence. These observations align with the results obtained on the synthetic datasets, where the Quadratic Hassanat loss demonstrated satisfactory calibration performance.

To comprehensively assess computational efficiency, we measured wall-clock training time alongside convergence behavior. Table 5 presents convergence epochs, per-epoch execution time, total training time, and estimated Floating Point Operations (FLOPs). H-Log and H-Log-N demonstrate the most efficient optimization trajectories, converging in 77.2 epochs compared to 78.7 for BCE. While current PyTorch implementations exhibit 12–15% per-epoch overhead relative to highly-optimized built-in BCE loss, the resulting total training times remain competitive (7.07–7.33 s vs. 6.27 s). The FLOPs analysis (~44 vs. ~46) confirms that this overhead is implementation-dependent rather than algorithmic, suggesting that custom-optimized kernels could achieve both fewer epochs *and* faster wall-clock time. Given that the current ~1 second difference is negligible relative to total research workflow time (data preparation, model selection, evaluation), the demonstrated benefits in calibration and robustness justify this modest computational cost.

**Table 5:** Computational efficiency analysis: convergence speed and training time comparison across loss functions.

**Loss function**	**Epochs to converge**	**Avg Epoch Time (ms)**	**Time to convergence (s)**	**Overhead vs BCE**	**Est. FLOPs/sample**
BCE	78.7 ± 28.1	129.4 ± 251.8	6.27 ± 10.58	Baseline	~46
Huber	81.5 ± 25.0	129.5 ± 252.0	6.57 ± 10.57	+0.1%	~8
MSE	84.6 ± 22.8	129.3 ± 252.1	6.67 ± 10.70	−0.1%	~2
LogCosh	83.6 ± 24.1	132.4 ± 257.7	6.88 ± 11.01	+2.3%	~25
H-Log	**77.2** **±26.7**	148.6 ± 289.2	7.07 ± 12.22	+14.8%	~44
H-Log-N	**77.2** **±25.2**	145.2 ± 282.6	7.33 ± 12.72	+12.2%	~44
L1	82.8 ± 22.5	130.9 ± 254.8	7.34 ± 12.46	+1.2%	~2
H^2^-N	84.5 ± 23.5	137.2 ± 267.0	7.50 ± 12.17	+6.0%	~11
H-Quad	84.8 ± 23.4	140.4 ± 273.9	7.67 ± 12.28	+8.5%	~11
H-Linear	81.9 ± 23.9	140.9 ± 274.6	7.90 ± 12.93	+8.9%	~10
Focal	87.9 ± 22.7	168.0 ± 324.3	8.53 ± 14.14	+29.8%	~60
H-N	86.3 ± 20.4	136.8 ± 266.6	9.24 ± 15.98	+5.7%	~10

To rigorously examine the difference between the best two performers, Quadratic Hassanat and BCE, ttest is applied to evaluate the difference. Although *p*-value is important to show the statistical difference between two methods, its results alone might be misleading in our case due to the lower samples size, number of folds for each dataset. Therefore, we paired each results with the cohen's d effect size to notice the practical difference between the compared methods.

As one can see from Tables 6, 7, Quadratic Hassanat shows good calibration that makes a noticeable difference in practice on several datasets, in terms of ECE, while on the rest of the datasets, there is no significant difference between the two loss function. For the log loss, the effect size was practically noticeable on some datasets, like Heart Disease, respectively, with a significant difference in *p*-value using the later metric, as also confirmed by the confidence intervals (see Table 7).

**Table 6:** Overall calibration performance: H-Quad vs BCE across 9 Datasets.

**Metric**	**H-Quad**	**BCE**	**Difference**	**95% CI**	**% Diff**	**Effect size**	***p*-value**	**Sig**	**Winner**
ECE	0.129 ± 0.074	0.134 ± 0.084	−0.005	[−0.012, 0.002]	−3.9%	Negligible	0.140	No	H-Quad
Brier score	0.163 ± 0.060	0.163 ± 0.059	0.000	[−0.002, 0.002]	0.0%	Negligible	0.961	No	BCE
Log loss	0.533 ± 0.191	0.553 ± 0.210	−0.020	[−0.032, −0.008]	−3.6%	Negligible	0.001^*^	Yes	H-Quad

Results based on 10-fold cross-validation to ensure robust statistical comparison. The symbol * inside the mentioned tables means the value indicates a significant statistical difference.

**Table 7:** Dataset-wise calibration performance: H-Quad vs BCE detailed analysis.

**Dataset**	**H-Quad**	**BCE**	**Diff**	**95% CI**	**Diff%**	**Effect**	**Effect size**	***p*-value**	**Sig**	**Winner**
**Ece**										
Adult income prediction	0.014	0.013	0.002	[−0.001, 0.004]	11.8	0.39	Small	0.262	No	BCE
Credit approval	0.109	0.126	−0.017	[−0.034, −0.001]	−13.8	−0.39	Small	0.041	^*^Yes	H–Quad
German credit numeric	0.162	0.174	−0.012	[−0.024, −0.001]	−6.9	−0.29	Small	0.043	^*^Yes	H-Quad
Haberman survival	0.191	0.185	0.006	[−0.045, 0.057]	3.4	0.11	Negligible	0.786	No	BCE
Heart Disease	0.179	0.182	−0.003	[−0.024, 0.019]	−1.4	−0.04	Negligible	0.792	No	H-Quad
Indian liver patient	0.134	0.123	0.011	[−0.012, 0.033]	8.7	0.31	Small	0.308	No	BCE
Liver disorder	0.193	0.229	−0.036	[−0.053, −0.019]	−15.7	−0.65	Medium	0.001	^*^Yes	H-Quad
Magic Gamma telescope	0.030	0.025	0.005	[0.001, 0.009]	19.0	0.75	Medium	0.029	^*^Yes	BCE
Pima Indians diabetes	0.145	0.148	−0.003	[−0.024, 0.018]	−1.9	−0.06	Negligible	0.769	No	H-Quad
**Brier score**										
Adult income prediction	0.101	0.100	0.000	[0.000, 0.001]	0.2	0.11	Negligible	0.084	No	BCE
Credit approval	0.124	0.125	−0.002	[−0.009, 0.006]	−1.3	−0.04	Negligible	0.656	No	H-Quad
German credit numeric	0.189	0.191	−0.002	[−0.008, 0.003]	−1.1	−0.08	Negligible	0.379	No	H-Quad
Haberman survival	0.200	0.198	0.001	[−0.008, 0.011]	0.6	0.03	Negligible	0.795	No	BCE
Heart disease	0.158	0.157	0.000	[−0.007, 0.008]	0.2	0.0	Negligible	0.936	No	BCE
Indian liver patient	0.181	0.183	−0.002	[−0.006, 0.002]	−1.1	−0.12	Negligible	0.276	No	H-Quad
Liver disorder	0.250	0.247	0.002	[−0.005, 0.009]	0.9	0.06	Negligible	0.491	No	BCE
Magic gamma telescope	0.087	0.087	0.000	[−0.002, 0.001]	−0.2	−0.05	Negligible	0.756	No	H-Quad
Pima Indians diabetes	0.183	0.181	0.003	[−0.008, 0.013]	1.4	0.08	Negligible	0.575	No	BCE
**Log loss**										
Adult income prediction	0.316	0.313	0.003	[0.001, 0.004]	0.8	0.32	Small	0.009	^*^Yes	BCE
Credit approval	0.472	0.522	−0.050	[−0.086, −0.015]	−9.7	−0.24	Small	0.010	^*^ Yes	H-Quad
German credit numeric	0.687	0.775	−0.088	[−0.123, −0.052]	−11.3	−0.62	Medium	0.000	^*^Yes	H-Quad
Haberman survival	0.594	0.591	0.003	[−0.018, 0.024]	0.5	0.02	Negligible	0.744	No	BCE
Heart disease	0.552	0.623	−0.071	[−0.101, −0.041]	−11.4	−0.31	Small	0.001	^*^Yes	H-Quad
Indian liver patient	0.527	0.531	−0.004	[−0.013, 0.006]	−0.7	−0.08	Negligible	0.415	No	H-Quad
Liver disorder	0.756	0.735	0.021	[−0.020, 0.062]	2.9	0.17	Negligible	0.269	No	BCE
Magic gamma telescope	0.289	0.287	0.002	[−0.001, 0.005]	0.7	0.15	Negligible	0.174	No	BCE
Pima Indians diabetes	0.601	0.596	0.004	[−0.055, 0.063]	0.7	0.03	Negligible	0.875	No	BCE

Results based on 10-fold cross-validation to ensure robust statistical comparison. The symbol * inside the mentioned tables means the value indicates a significant statistical difference.

These results indicate that, Quadratic Hassanat is better choice when the calibration is favorable over classification metrics as it is at least performs similar to BCE, on average, based on the experiments conducted in this study.

BCE is the first option one can think of when performing binary classification task, when the classification metrics are concerned. However, this study shows that the proposed H-N variant, gives better classification metrics, on the utilized datasets. To confirm this another statistical test and effect size analysis is conducted between H-N and BCE to compare their classification metrics performance.

Table 8 presents a comprehensive statistical comparison of H-N and BCE performance across nine datasets using precision, recall, and F1-score metrics. The results reveal nuanced performance patterns with only two datasets showing statistically significant differences. BCE demonstrates statistically significant superiority on Magic Gamma Telescope across all metrics with large effect sizes: precision (−1.7%, *p* = 0.017, Cohen's d = −0.92), recall (−2.0%, *p* = 0.015, d = −0.88), and F1-score (−1.8%, *p* = 0.001, d = −1.22), and on Adult Income Prediction for recall (−4.1%, *p* = 0.023, d = −0.98) and F1-score (−2.4%, *p* = 0.004, d = −1.08). Despite lacking statistical significance, H-N shows consistent performance advantages on medical diagnosis datasets, particularly Indian Liver Patient with improvements in precision (+0.2%), recall (+16.2%), and F1-score (+8.8%), and Heart Disease with gains in precision (+2.2%), recall (+1.6%), and F1-score (+2.0%). The most pronounced non-significant differences favoring H-N appear on Haberman Survival, where precision increases by 55.8% (medium effect size, d = 0.65) and F1-score by 49.3% (medium effect size, d = 0.56), though *p*-values remain above 0.05. Overall, H-N wins on precision for six of nine datasets while BCE wins on recall for five of nine datasets, though the limited number of statistically significant results suggests that the choice between H-N and BCE may depend on specific application requirements rather than clear statistical superiority.

**Table 8:** Dataset-wise classification performance: H-N vs BCE.

**Dataset**	**H-N**	**BCE**	**Diff**	**95% CI**	**Diff%**	**Effect**	**Effect size**	***p*-value**	**Sig**	**Winner**
**Precision**										
Adult Income Prediction	0.741	0.741	0.001	[−0.011, 0.013]	0.1	0.03	Negligible	0.915	No	H-N
Credit Approval	0.885	0.873	0.013	[−0.018, 0.043]	1.4	0.21	Small	0.373	No	H-N
German Credit Numeric	0.604	0.613	−0.009	[−0.043, 0.025]	−1.5	−0.14	Negligible	0.566	No	BCE
Haberman Survival	0.483	0.310	0.173	[−0.049, 0.395]	55.8	0.65	Medium	0.112	No	H-N
Heart Disease	0.840	0.822	0.018	[−0.006, 0.042]	2.2	0.17	Negligible	0.125	No	H-N
Indian Liver Patient	0.473	0.472	0.001	[−0.065, 0.067]	0.2	0.01	Negligible	0.979	No	H-N
Liver Disorder	0.628	0.591	0.038	[−0.037, 0.112]	6.3	0.28	Small	0.285	No	H-N
Magic Gamma Telescope	0.888	0.904	−0.015	[−0.027, −0.003]	−1.7	−0.92	Large	0.017	^*^Yes	BCE
Pima Indians Diabetes	0.658	0.636	0.021	[−0.041, 0.084]	3.4	0.25	Small	0.459	No	H-N
**Recall**										
Adult Income Prediction	0.571	0.596	−0.025	[−0.045, −0.004]	−4.1	−0.98	Large	0.023	^*^Yes	BCE
Credit approval	0.864	0.862	0.003	[−0.018, 0.023]	0.3	0.04	Negligible	0.774	No	H-N
German credit numeric	0.573	0.603	−0.030	[−0.078, 0.018]	−5.0	−0.42	Small	0.193	No	BCE
Haberman survival	0.293	0.206	0.088	[−0.059, 0.234]	42.6	0.48	Small	0.209	No	H-N
Heart disease	0.853	0.840	0.013	[−0.036, 0.063]	1.6	0.15	Negligible	0.555	No	H-N
Indian liver patient	0.402	0.346	0.056	[−0.115, 0.227]	16.2	0.38	Small	0.479	No	H-N
Liver disorder	0.457	0.464	−0.007	[−0.062, 0.048]	−1.5	−0.04	Negligible	0.778	No	BCE
Magic Gamma telescope	0.734	0.749	−0.015	[−0.026, −0.004]	−2.0	−0.88	Large	0.015	^*^Yes	BCE
Pima Indians diabetes	0.586	0.616	−0.030	[−0.087, 0.027]	−4.8	−0.32	Small	0.270	No	BCE
**F1**										
Adult income prediction	0.645	0.660	−0.016	[−0.025, −0.006]	−2.4	−1.08	Large	0.004	^*^Yes	BCE
Credit approval	0.873	0.866	0.007	[−0.012, 0.026]	0.8	0.13	Negligible	0.429	No	H-N
German credit numeric	0.586	0.605	−0.019	[−0.051, 0.013]	−3.1	−0.34	Small	0.214	No	BCE
Haberman survival	0.360	0.241	0.119	[−0.056, 0.294]	49.3	0.56	Medium	0.159	No	H-N
Heart disease	0.843	0.827	0.017	[−0.018, 0.051]	2.0	0.22	Small	0.302	No	H-N
Indian liver patient	0.419	0.385	0.034	[−0.089, 0.156]	8.8	0.29	Small	0.547	No	H-N
Liver Disorder	0.519	0.510	0.009	[−0.041, 0.059]	1.8	0.07	Negligible	0.683	No	H-N
Magic Gamma telescope	0.804	0.819	−0.015	[−0.022, −0.008]	−1.8	−1.22	Large	0.001	^*^Yes	BCE
Pima Indians diabetes	0.615	0.622	−0.007	[−0.060, 0.045]	−1.2	−0.11	Negligible	0.764	No	BCE

Results based on 10-fold cross-validation to ensure robust statistical comparison. The symbol * inside the mentioned tables means the value indicates a significant statistical difference.

This opens the door to further investigate the proposed loss function and its variants in relation to dataset characteristics, particularly examining how specific data properties, such as class imbalance ratios, feature complexity, biomarker interactions, and domain, specific decision boundaries, influence the relative performance of different loss functions. Future research could systematically evaluate Hassanat loss and its variants across datasets with varying imbalance levels, feature types (continuous biomarkers vs. categorical variables), and clinical contexts to establish guidelines for optimal loss function selection based on dataset characteristics. Additionally, investigating the theoretical properties of H-N that enable better handling of complex feature interactions and minority class detection could inform the development of adaptive loss functions that automatically adjust to dataset-specific challenges, ultimately advancing personalized machine learning approaches for medical diagnosis and other critical classification tasks.

## 5 Conclusion

This study introduced novel loss function variants based on the Hassanat distance metric for binary classification tasks. Through comprehensive evaluation on both synthetic and real-world datasets, we demonstrated that these functions offer compelling alternatives to traditional loss functions like Binary Cross-Entropy, particularly in scenarios involving noisy data and outliers. The quadratic variant (H-Quad) consistently achieved superior calibration performance with the lowest Expected Calibration Error values, while the linear variant with centrality-based normalization (H-N) excelled in classification metrics. Most importantly, the Hassanat variants demonstrated notable robustness to outliers and noise while requiring fewer epochs to converge, critical advantages in real, world applications where data quality and computational efficiency matter.

However, our findings reveal important trade-offs that practitioners must consider. While H-N achieved the best classification performance, it showed poorer calibration compared to other variants, suggesting careful selection based on specific application requirements. Several limitations should be acknowledged: our evaluation focused exclusively on binary classification, used relatively simple architectures, and tested on only nine real-world datasets. The theoretical understanding of convergence properties remains incomplete, and the computational overhead of complex variants needs more detailed analysis.

Based on our findings, we recommend H-N for applications prioritizing classification performance, especially with imbalanced datasets, and H-Quad for scenarios requiring well-calibrated probabilities like medical diagnosis or risk assessment. For noisy or outlier-prone datasets, any Hassanat variant will likely outperform traditional losses, with H-Quad offering the best balance of robustness and calibration. Future research should focus on extending these functions to multi-class classification, developing rigorous theoretical analysis of convergence properties, and investigating performance with modern deep learning architectures, where the proposed loss function might have better properties than existing loss function. Additionally, exploring adaptive variants that adjust during training, computational optimizations for large-scale applications, and domain-specific evaluations could further enhance their practical value.

The consistent performance improvements across diverse datasets suggest that Hassanat-based loss functions represent a valuable addition to the machine learning ecosystem. As the field continues addressing increasingly complex and noisy real-world data, robust and well-calibrated loss functions like these will become even more important, providing a solid foundation for continued exploration of distance-based approaches to loss function design.

## Data Availability

The datasets are publicly available and downloaded from https://github.com/MachineLearningBCAM/Datasets.
